# Educational inequalities in premature mortality by region in the Belgian population in the 2000s

**DOI:** 10.1186/s13690-017-0212-x

**Published:** 2017-10-16

**Authors:** Françoise Renard, Brecht Devleesschauwer, Sylvie Gadeyne, Jean Tafforeau, Patrick Deboosere

**Affiliations:** 1 Department of Public Health and Surveillance, Scientific Institute of Public Health (WIV-ISP), Rue Juliette Wytsmanstraat 14, 1050 Brussels, Belgium; 2 Interface Demography, Section Social Research, Vrije Universiteit Brussels, Brussels, Belgium

**Keywords:** Health inequalities, Educational inequalities, Premature mortality, Belgium

## Abstract

**Background:**

In Belgium, socio-economic inequalities in mortality have long been described at country-level. As Belgium is a federal state with many responsibilities in health policies being transferred to the regional levels, regional breakdown of health indicators is becoming increasingly relevant for policy-makers, as a tool for planning and evaluation. We analyzed the educational disparities by region for all-cause and cause-specific premature mortality in the Belgian population.

**Methods:**

Residents with Belgian nationality at birth registered in the census 2001 aged 25–64 were included, and followed up for 10 years though a linkage with the cause-of-death database. The role of 3 socio-economic variables (education, employment and housing) in explaining the regional mortality difference was explored through a Poisson regression. Age-standardised mortality rates (ASMRs) by educational level (EL), rate differences (RD), rate ratios (RR), and population attributable fractions (PAF) were computed in the 3 regions of Belgium and compared with pairwise regional ratios. The global PAFs were also decomposed into the main causes of death.

**Results:**

Regional health gaps are observed within each EL, with ASMRs in Brussels and Wallonia exceeding those of Flanders by about 50% in males and 40% in females among Belgian. Individual SE variables only explained up to half of the regional differences. Educational inequalities were also larger in Brussels and Wallonia than in Flanders, with RDs ratios reaching 1.8 and 1.6 for Brussels versus Flanders, and Wallonia versus Flanders respectively; regional ratios in relative inequalities (RRs and PAFs) were smaller. This pattern was observed for all-cause and most specific causes of premature mortality. Ranking the cause-specific PAFs revealed a higher health impact of inequalities in causes combining high mortality rate and relative inequality, with lung cancer and ischemic heart disease on top for all regions and both sexes. The ranking showed few regional differences.

**Conclusions:**

For the first time in Belgium, educational inequalities were studied by region. Among the Belgian, educational inequalities were higher in Brussels, followed by Wallonia and Flanders. The region-specific PAF decomposition, leading to a ranking of causes according to their population-level impact on overall inequality, is useful for regional policy-making processes.

**Electronic supplementary material:**

The online version of this article (doi:10.1186/s13690-017-0212-x) contains supplementary material, which is available to authorized users.

## Background

Socio-economic (SE) inequalities in health are a well-known fact, and reducing them is a public health priority [1–3] requiring careful monitoring [4]. In Belgium, SE inequalities in mortality, life and health expectancies have mostly been studied at country-level [5–8]. In a previous study [9], we focused on educational inequalities in all-cause and cause-specific premature mortality and their evolutions from the 1990s to the 2000s in Belgium as a whole. However, country averages for health outcomes hide important within-country variations. As Belgium is a federal state with more and more responsibilities in health policies transferred to the regional level – i.e., the Flemish, the Brussels Capital, and the Walloon Region - the regional breakdown of health indicators is highly relevant for policy-makers, not only as a possible mirror of different risk factor patterns but also as a tool for planning and evaluation.

While geographical disparities in mortality have been long and abundantly studied [10–17], up to now only two studies analyzed both the regional and SE disparities in mortality, yet with an aim to explain the geographical pattern of all-cause mortality [18, 19].

Building on our previous studies [9, 10, 17] that described the regional and educational premature mortality gaps in the 2000s, this papers aims to assess regional disparities in all-cause and cause-specific premature mortality in the Belgian population.

The aim of this study is triple. First, we want to estimate which proportion of the regional disparities in premature mortality in the 2000s can be explained by individual socio-economic characteristics. Secondly, we aim to compare the relative, absolute and population-level educational inequalities in mortality by region and thirdly, estimate and rank, in each region, the potential population-level impact that would result from reducing inequalities in a selection of twelve important avoidable causes of death. The identification of these causes of death with the largest population-level inequalities is of particular public health relevance, as this information can help to set priorities in policies tackling health inequalities. To achieve this ranking, we perform a decomposition of the population attributable fraction (PAF) by causes of death.

## Materials and methods

### Data

The data used in the current study were obtained by linking a) the 2001 Belgian population census, b) the National Population Register, and c) the causes of death database for the period 2001–2011 [5, 20]. Although a more recent census has been held in 2011, it has not been used as the databases linkages have not been performed yet.

The study population comprised all persons aged 25–64 at census, officially residing in Belgium, and having the Belgian nationality at birth (*N* = 4,556,830 persons). First generation migrants (operationally defined as not having the Belgian nationality at birth) made up 17.5% of the census 2001 in the age group 25–64, with substantial differences by region (i.e., 9%, 50% and 22% in Flanders, Brussels and Wallonia, respectively). First generation migrants (henceforth referred to as “Migrants”) experience lower mortality rates than people with Belgian nationality at birth (henceforth referred to as “Belgians”) [21, 22], for each educational level and each region (Additional file 1: Table S1). In this study, we chose to focus on the Belgian population, which has been exposed since birth to the life conditions and health policies prevailing in Belgium. Furthermore, migrants represent a highly inhomogeneous population with various ethnic and socio-economic backgrounds and would therefore deserve a careful study by origin and ethnicity. The follow-up consisted of a 10 years period after the census (Table 1).

**Table 1:** Number of persons, of person-years of follow up and of deaths included in the follow up by region. Distribution by age, education level housing score and employment status, people of Belgian origin aged 25–64 at census, Belgium, follow up 2001–11

	Flanders	Brussels	Wallonia	Total
N	2,922,767	260,619	1,373,444	4,556,830
%	64.14	5.72	30.14	100.00
Person-years of follow up	27,819,080	2,430,134	12,949,874	43,199,088
Number of deaths	107,030	14,280	72,916	194,226
Gender				
* Male (%)*	50.4	48.5	49.6	
* Female (%)*	49.6	51.5	50.4	
Education				
* Low (%)*	36.8	29.2	39.0	
* Mid (%)*	31.3	21.4	26.7	
* High (%)*	27.5	40.2	27.1	
* Missing (%)*	4.4	9.2	7.1	
Housing				
* Tenant/Low comfort (%)*	6.8	11.9	9.6	
* Tenant/Mid comfort (%)*	5.0	16.5	6.7	
* Tenant/High comfort (%)*	7.9	15.4	7.0	
* Owner/Low comfort (%)*	17.8	5.5	18.4	
* Owner/Mid comfort (%)*	6.9	8.0	7.9	
* Owner/High comfort (%)*	50.7	31.2	42.7	
* Missing (%)*	4.9	11.5	7.6	
Employment status				
* Working (incl student) (%)*	69.5	65.5	62.6	
* Unemployed (%)*	3.6	7.6	8.8	
* Retired (%)*	10.3	9.4	10.0	
* Non-working (%)*	13.8	10.7	14.1	
* Missing (%)*	2.7	6.7	4.5	

This study included people aged 25–64 at census who were followed up for 10 years, except for the age group 60–64 for which the follow up time was censored at 70 years. Mortality before the age of 70 was defined as “premature mortality”, for the sake of simplicity also referred to as “mortality” in the manuscript.

To assess the contribution of the individual socioeconomic (SE) status to regional mortality differences, we used a set of three SE variables: educational level (EL), employment status and housing status. EL was categorized according to the highest obtained degree using the International Standard Classification of Education (ISCED), version 1997 [23]. Three categories were created: lower secondary education or less (ISCED 0–2; “low”), higher secondary education (ISCED 3–4; “mid”) and tertiary education (ISCED 5–6; “high”). The employment status was classified into 4 classes: “working, including students”, “unemployed” (designating people getting unemployment allocations), “retired”, and “not working, other”. This last group, although quite heterogeneous with respect to the reasons for non-working, contains, in men, a large proportion of people with health problems, which probably reflect a health selection in the labor market (people in good health are more prone to work). The housing status was based on information regarding tenure status and housing quality. This variable consisted of six categories (low-, mid- and high-comfort tenants and low-, mid- and high comfort owners) and was measured at the household level [24, 25]. It can be considered as a good proxy for wealth.

To compare SE inequalities by region, we focused on educational level only. Educational attainment is a relatively stable measure of SE position, as usually achieved early in adulthood, and is usually of rather good quality [26].

Causes of death were classified according to the International Classification of Diseases (ICD), version 10 [27]. All-cause premature mortality was divided into two categories (avoidable and non-avoidable mortality) according to the recent UK Office of Statistics definition of avoidable mortality [28] also adopted by Eurostat [29]. In addition, we divided the total premature mortality in four broad groups of causes of deaths (circulatory diseases, cancers, other natural causes of deaths and external causes), and further analyzed 12 avoidable causes of death with a high burden in Belgian society (lung cancer, lip, oral cavity and pharynx cancer, colorectal cancer, liver cancer, ischemic heart diseases (IHD), cerebrovascular diseases that were grouped with hypertension (HTA) as usually recommended [30, 31], alcohol related deaths, diabetes, chronic obstructive pulmonary diseases (COPD), suicide, and transport accidents). In women, breast cancer was analyzed as well. The corresponding ICD10 codes are shown in Additional file 1: Table S2. Analyses were performed separately for men and women as they have very different mortality levels.

### Analyses

#### Premature mortality rates by region and individual socio-economic characteristics

We first computed in each region age-standardized mortality rates (ASMR) by EL, sex and cause of death, using the European population as reference population [32].

Rates were expressed per 100,000 person-years (PYs); the PYs were calculated as the sum across all people included in the census cohort, of individual times between census date and either date of death, emigration date or last day of the study. People having emigrated were censored at emigration date. To take into account the ageing process during follow-up, age was introduced as a time-varying variable. Standard errors on rates were computed in Stata assuming a binomial distribution.

We first focused on regional rates, comparing EL-specific all-cause ASMRs between each pair (i, j) of regions (i.e., Brussels versus Flanders, Wallonia versus Flanders and Wallonia versus Brussels). For each EL (x = 1, … , 4), we calculated between-region rate differences, $\left( ASM{R}_{i,x}- ASM{R}_{j,x}\right)$, as well as between-region rate excesses, $\left(\left[{ASMR}_{i,x}/{ASMR}_{j,x}\right]-1\right)$, and used a z-test to assess statistical significance [33, 34].

In order to assess together the regional pattern and the influence of individual SE variables on this pattern, we fitted three different Poisson regression models (Table 2). In the first model, mortality was simply regressed against region, controlling for current age. In three variants of an intermediate model (models 2a, 2b, and 2c), each SE variable – EL, employment status and housing status – was added separately. As all three SE variables revealed to have a significant effect on overall mortality, they were introduced simultaneously in a multivariable model (model 3). This third model allowed assessing to which extent the individual SE level could explain the regional gaps in mortality. Cases with missing information were introduced as specific classes in the analyses.

**Table 2:** Premature mortality: Rate Ratios and *p*-values for different Poisson regression models including age at inclusion in the cohort, region of residence, and SE variables (education level, employment status, housing score). People of Belgian nationality at birth aged 25–64 at census, Belgium, follow up 2001–11

		Model 1	*p value*	Model 2a	*p value*	Model 2b	*p value*	Model 2c	*p value*	Model 3	*p value*
Men											
Curage		1.088	*<0.001*	1.083	*<0.001*	1.074	*<0.001*	1.092	*<0.001*	1.078	*<0.001*
Region	Bxl	1.539	*<0.001*	1.579	*<0.001*	1.404	*<0.001*	1.222	*<0.001*	1.254	*<0.001*
	Wal	1.543	*<0.001*	1.513	*<0.001*	1.452	*<0.001*	1.429	*<0.001*	1.390	*<0.001*
Education	Low			1.967	*<0.001*					1.373	*<0.001*
	Mid			1.470	*<0.001*					1.258	*<0.001*
	Missing			3.046	*<0.001*					1.469	*<0.001*
Employment	Unempl.					2.582	*<0.001*			1.992	*<0.001*
	Retired					1.598	*<0.001*			1.455	*<0.001*
	Non working, other					3.495	*<0.001*			2.688	*<0.001*
	Missing					3.290	*<0.001*			2.078	*<0.001*
Housing	Ten./Low comf							2.853	*<0.001*	2.071	*<0.001*
	Ten./Mid comf							2.966	*<0.001*	2.172	*<0.001*
	Ten./High comf							1.595	*<0.001*	1.477	*<0.001*
	Owner/Low comf							1.642	*<0.001*	1.408	*<0.001*
	Owner/Mid comf							1.599	*<0.001*	1.374	*<0.001*
	Missing							2.859	*<0.001*	1.852	*<0.001*
Women											
Curage		1.085	*<0.001*	1.077	*<0.001*	1.068	*<0.001*	1.086	*<0.001*	1.071	*<0.001*
Region	Bxl	1.491	*<0.001*	1.555	*<0.001*	1.586	*<0.001*	1.238	*<0.001*	1.346	*<0.001*
	Wal	1.401	*<0.001*	1.393	*<0.001*	1.391	*<0.001*	1.310	*<0.001*	1.309	*<0.001*
Education	Low			1.718	*<0.001*					1.204	*<0.001*
	Mid			1.381	*<0.001*					1.155	*<0.001*
	Missing			2.697	*<0.001*					1.469	*<0.001*
Employment	Unempl.					1.802	*<0.001*			1.516	*<0.001*
	Retired					1.785	*<0.001*			1.593	*<0.001*
	Non working, other					2.193	*<0.001*			1.950	*<0.001*
	Missing					2.836	*<0.001*			1.888	*<0.001*
Housing	Ten./Low comf							2.422	*<0.001*	2.122	*<0.001*
	Ten./Mid comf							2.469	*<0.001*	2.200	*<0.001*
	Ten./High comf							1.509	*<0.001*	1.501	*<0.001*
	Owner/Low comf							1.477	*<0.001*	1.361	*<0.001*
	Owner/Mid comf							1.416	*<0.001*	1.326	*<0.001*
	Missing							2.521	*<0.001*	2.009	*<0.001*

#### Calculation of educational inequalities by region

Measuring inequalities is a complex issue [35–37]. Indeed, health inequality can be measured from several perspectives [38] – for instance a simple comparison of two social groups versus a population-wide perspective, or the measurement of absolute versus relative inequalities. As each inequality measure captures only a partial aspect of inequality, it is recommended to use a set of complementary indices [36, 38, 39], preferably including simple absolute and relative pairwise measures along with measures summarizing inequalities across the whole population.

Within each region, three inequality indices were calculated for all-cause mortality, for each broad cause group and for each selected cause of premature mortality: namely, two pairwise inequality indices, i.e., the low-versus-high EL absolute rate difference (RD), calculated as ASMR_low EL_ – ASMR_High EL_ and rate ratio (RR), calculated as ASMR_low EL_ / ASMR_High EL_, and one composite measure, i.e., the population attributable fraction (PAF), measuring the population impact of inequality on mortality. The global PAF indicates which fraction of all deaths would have been avoided (in people aged 25–64 at baseline) if the mortality of the total population were equal to the one observed in the highest EL. The global PAF is calculated as:

$$ \frac{\mathrm{ASMR}\  \mathrm{in}\  \mathrm{the}\  \mathrm{total}\  \mathrm{population}-\mathrm{ASMR}\  \mathrm{in}\  \mathrm{the}\  \mathrm{highest}\  EL}{\mathrm{Overall}\  \mathrm{mortality}\  \mathrm{in}\  \mathrm{the}\  \mathrm{total}\  \mathrm{population}} $$

We also estimated the specific contributions of the 12 main avoidable causes of death to the PAF, by calculating the cause-specific PAFs. This measure indicates which fraction of all deaths would have been avoided (in people aged 25–64 at baseline) if the mortality from this cause in the whole population were equal to the one observed in the highest EL. The cause-specific PAFs are calculated as:

$$ \frac{\mathrm{ASMR}\ \mathrm{for}\ \mathrm{a}\ \mathrm{specific}\  \mathrm{cause}\  \mathrm{in}\  \mathrm{the}\  \mathrm{total}\  \mathrm{population}-\mathrm{ASMR}\ \mathrm{for}\ \mathrm{a}\ \mathrm{specific}\  \mathrm{cause}\  \mathrm{in}\  \mathrm{the}\  \mathrm{highest}\ \mathrm{EL}\ }{\mathrm{Overall}\  \mathrm{mortality}\  \mathrm{in}\  \mathrm{the}\  \mathrm{total}\  \mathrm{population}} $$

Standard errors on the PAFs were calculated by a Monte Carlo simulation approach [40].

#### Comparison of the magnitude of inequalities between the regions

The relative differences in region-specific educational RDs, RRs and PAFs were measured through three ratios (i.e. one for Brussels-Flanders, for Wallonia-Flanders and for Wallonia-Brussels), each calculated for all-cause and for cause-specific mortality. For instance, the Brussels-Flanders RD ratio was computed by dividing the educational RD in Brussels by the educational RD in Flanders; the Brussels-Flanders RR ratio was computed by dividing the educational RR in Brussels by the educational RR in Flanders. The statistical significance of these comparisons was calculated according to Altman’s method [41].

Analyses were performed in Stata version 14, and in R version 3.4.0.

## Results

### Basic characteristics of the population by region

Table 1 reveals clear regional differences with respect to individual SE features in our study population. Brussels is characterized by a higher proportion of higher educated individuals compared with the two other regions, whereas Flanders shows a higher housing score than Wallonia and Brussels (with a smaller proportion of owners in Brussels) and a lower rate of unemployment. It is also noteworthy that the proportion of missing values for the SE variables is lowest in Flanders.

### Premature mortality rates by region and socio-economic characteristics

**Fig. 1:**
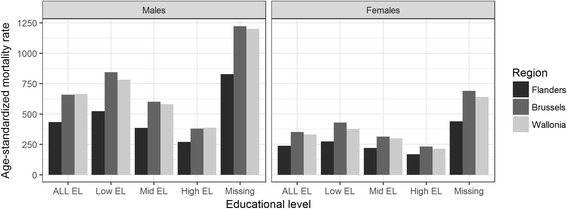
Age-standardized all-cause premature mortality rates by region and by educational levels. People of Belgian origin aged 25–64 at census, Belgium, follow up 2001–11

Figure 1 shows the total and the EL-specific ASMRs by region for the total Belgian population (detailed numbers in Additional file 1: Table S1, middle part). In the Walloon region, the ASMR was 54% higher than in Flanders among males and 40% higher among females, while in Brussels the ASMR was respectively 52% and 48% higher compared with Flanders. Similarly, all EL-specific ASMRs were higher in Wallonia and Brussels than in Flanders both among men and women, particularly in the lowest EL. In men, ASMRs were higher in Brussels than in Wallonia for the low and mid ELs, but lower than in the Walloon Region for the highest EL. In women, the ASMRs were higher in Brussels than in Wallonia for all ELs. Cause-specific ASMRs by region and regional differences are displayed in Additional file 1: Table S3. The respective effects of three individual SE variables on the regional effect were explored through several Poisson models and summarized in Table 2. Compared to the first basic model, containing region and age only, models 2a, 2b and 2c revealed that all three SE variables had an important and statistically significant effect on the mortality rates (RR between 1.5 and 3.2). In addition, the SE variables had a significant impact on regional mortality differences. This confounding effect varied by SE dimension and gender: 1) after adjusting for education (model 2a), the RR for Brussels versus Flanders increased slightly (which was expected given the higher proportion of the higher educated in Brussels); 2) adjusting for employment status (model 2b) reduced regional differences in males both in Brussels and the Walloon region compared with Flanders (the RR decreased from 1.54 to 1.40 and from 1.53 to 1.40 respectively), but slightly increased the regional RRs in women; 3) introducing the housing score (model 2c) substantially decreased (up to 50% in Brussels) the regional RRs both among men and women. Model 3 showed the combined effect of all three SE variables on regional differences, that all remained significant.

### Inequality indices by region

#### Educational rate differences (RDs) and rate ratios (RRs) by region

Absolute inequalities (RDs): Tables 3 and 4 (left part) show the pairwise educational RDs by region, for all-cause mortality, broad classes of causes and cause-specific premature mortality for men and women. In men, the all-cause and most cause-specific educational RDs were very important in all three regions; in women, the RDs were somewhat smaller than in men, given the lower ASMRs.

**Table 3:** All cause and cause-specific premature mortality by region: low and mid versus high educational level rate differences and rate ratio, males of Belgian origin aged 25–64 at census, Belgium, follow up 2001–11

			*Rate Difference*									*Rate Ratio*								
	*Males*		Fla	Bxl	Wal	RD ratio Bxl - Fla	*p*	RD ratio Wal - Fla	*p*	RD ratio Wal -Bxl	*p*	Fla	Bxl	Wal	RR ratio Bxl- Fla	*p*	RR ratio Wal -Fla	*p*	RR ratio Wal -Bxl	*p*
*All-Cause*	*ALL CAUSES*	*Low*	252.0	464.2	394.7	1.84	***	1.57	***	0.85	***	1.9	2.2	2.0	1.15	***	1.04	*	0.91	**
		*Mid*	116.1	221.1	193.8	1.90	***	1.67	***	0.88	ns	1.4	1.6	1.5	1.11	**	1.05	*	0.95	ns
	*Avoidable*	*Low*	195.3	340.9	289.6	1.75	***	1.48	***	0.85	**	2.1	2.4	2.2	1.14	**	1.01	ns	0.89	**
		*Mid*	92.0	167.2	139.6	1.82	***	1.52	***	0.83	bl	1.5	1.7	1.6	1.11	*	1.01	ns	0.92	bl
	*Not Avoidable*	*Low*	56.7	123.3	105.1	2.17	***	1.85	***	0.85	bl	1.6	1.9	1.8	1.19	**	1.12	***	0.94	ns
		*Mid*	24.0	53.8	54.2	2.24	**	2.26	***	1.01	ns	1.3	1.4	1.4	1.11	ns	1.12	**	1.01	ns
*Broad Classes*	*ALL CANCERS*	*Low*	79.4	140.8	109.4	1.77	***	1.38	***	0.78	**	1.7	2.0	1.8	1.21	***	1.06	*	0.88	*
		*Mid*	36.7	69.9	48.3	1.91	**	1.32	*	0.69	bl	1.3	1.5	1.4	1.15	*	1.02	ns	0.89	bl
	*ALL CIRCULAT. DIS.*	*Low*	56.7	106.9	86.2	1.89	***	1.52	***	0.81	**	2.0	2.4	2.2	1.23	**	1.08	*	0.88	bl
		*Mid*	26.1	50.6	43.5	1.94	**	1.66	***	0.86	ns	1.5	1.7	1.6	1.16	bl	1.09	bl	0.94	ns
	*OTH.NAT.DEATHS*	*Low*	60.4	148.2	132.2	2.45	***	2.19	***	0.89	ns	2.0	2.3	2.2	1.14	*	1.08	*	0.94	ns
		*Mid*	23.9	73.3	64.8	3.07	***	2.71	***	0.88	ns	1.4	1.7	1.6	1.17	*	1.13	**	0.96	ns
	*EXTERNAL CAUSES*	*Low*	55.5	68.3	66.8	1.23	ns	1.20	*	0.98	ns	2.5	2.2	2.1	0.89	ns	0.83	**	0.94	ns
		*Mid*	29.4	27.2	37.2	0.93	ns	1.27	*	1.36	ns	1.8	1.5	1.6	0.83	bl	0.89	*	1.08	ns
*Detailed*	*Lung Ca*	*Low*	44.8	62.2	58.8	1.39	**	1.31	***	0.95	ns	2.5	2.7	2.6	1.07	ns	1.05	ns	0.97	ns
		*Mid*	19.6	27.0	25.4	1.38	ns	1.29	bl	0.94	ns	1.6	1.7	1.7	1.05	ns	1.02	ns	0.98	ns
	*Colorectal Ca*	*Low*	3.1	9.7	2.1	3.11	*	0.69	ns	0.22	**	1.2	1.8	1.2	1.43	*	0.93	ns	0.65	*
		*Mid*	3.0	4.1	1.3	1.35	ns	0.43	ns	0.32	ns	1.2	1.3	1.1	1.07	ns	0.89	ns	0.83	ns
	*Lip-Or.Cav-Phar.Ca*	*Low*	5.1	12.3	8.3	2.40	**	1.63	**	0.68	ns	2.1	3.0	2.5	1.47	bl	1.23	ns	0.84	ns
		*Mid*	2.2	6.0	4.1	2.75	ns	1.84	bl	0.67	ns	1.5	2.0	1.7	1.37	ns	1.20	ns	0.87	ns
	*LiverCancer*	*Low*	1.0	3.8	2.3	3.80	ns	2.28	ns	0.60	ns	1.3	1.7	1.4	1.31	ns	1.08	ns	0.82	ns
		*Mid*	1.2	5.1	1.7	4.22	ns	1.39	ns	0.33	ns	1.4	1.9	1.3	1.42	ns	0.95	ns	0.67	ns
	*Prostate Ca*	*Low*	1.1	−1.3	2.4	−1.24	ns	2.23	ns	−1.80	*	1.2	0.8	1.4	0.66	ns	1.20	ns	1.81	*
		*Mid*	0.4	0.6	1.2	1.61	ns	3.00	ns	1.87	ns	1.1	1.1	1.2	1.02	ns	1.13	ns	1.11	ns
	*Isc.Heart Dis.*	*Low*	28.8	50.4	45.4	1.75	***	1.57	***	0.90	ns	2.1	2.4	2.3	1.14	ns	1.09	ns	0.96	ns
		*Mid*	12.8	22.2	20.5	1.73	bl	1.60	**	0.92	ns	1.5	1.6	1.6	1.08	ns	1.06	ns	0.98	ns
	*Cer.vasc.Dis/HTA*	*Low*	8.7	16.0	14.4	1.84	*	1.66	***	0.90	ns	1.9	2.5	2.0	1.32	ns	1.09	ns	0.83	ns
		*Mid*	3.5	13.2	7.7	3.71	*	2.17	*	0.58	ns	1.4	2.2	1.6	1.63	*	1.15	ns	0.71	ns
	*C.o.p.d.*	*Low*	12.8	26.0	27.7	2.03	***	2.16	***	1.06	ns	3.9	4.5	5.1	1.17	ns	1.33	*	1.13	ns
		*Mid*	3.5	9.2	7.9	2.61	bl	2.25	**	0.86	ns	1.8	2.2	2.2	1.26	ns	1.22	ns	0.97	ns
	*Alc. Rel Dth*	*Low*	8.1	31.6	21.1	3.91	***	2.62	***	0.67	*	1.7	2.3	1.8	1.37	*	1.04	ns	0.76	*
		*Mid*	4.7	23.7	16.6	5.06	***	3.55	***	0.70	ns	1.4	2.0	1.6	1.42	*	1.14	ns	0.80	ns
	*Diabetes*	*Low*	2.8	9.0	6.9	3.23	**	2.46	***	0.76	ns	2.2	3.6	2.5	1.65	ns	1.15	ns	0.70	ns
		*Mid*	1.1	1.7	4.3	1.57	ns	4.04	**	2.57	ns	1.5	1.5	2.0	1.02	ns	1.34	ns	1.31	ns
	*Mental/neurol. Dis*	*Low*	9.3	28.6	21.9	3.06	***	2.35	***	0.77	ns	2.0	3.4	2.2	1.71	**	1.11	ns	0.65	*
		*Mid*	3.7	17.9	11.4	4.88	***	3.13	***	0.64	ns	1.4	2.5	1.6	1.80	**	1.17	ns	0.65	*
	*Ill Defined*	*Low*	4.3	16.2	12.8	3.79	**	2.99	***	0.79	ns	1.7	2.3	2.1	1.35	ns	1.26	*	0.93	ns
		*Mid*	2.1	6.1	6.9	2.95	ns	3.32	**	1.12	ns	1.3	1.5	1.6	1.11	ns	1.20	ns	1.08	ns
	*Suicide*	*Low*	28.7	34.9	31.9	1.21	ns	1.11	ns	0.92	ns	2.3	2.0	2.0	0.87	ns	0.86	*	0.99	ns
		*Mid*	14.6	16.4	18.6	1.12	ns	1.27	ns	1.13	ns	1.7	1.5	1.6	0.88	ns	0.94	ns	1.07	ns
	*Transport Acc.*	*Low*	14.0	7.1	17.4	0.51	*	1.25	ns	2.44	**	2.7	2.1	2.1	0.76	ns	0.78	bl	1.03	ns
		*Mid*	9.0	2.7	10.5	0.30	*	1.17	ns	3.90	**	2.1	1.4	1.7	0.66	ns	0.80	bl	1.20	ns

Significance codes: 0 ‘***’ 0.001 ‘**’ 0.01 ‘*’ 0.05 ‘bl’ [borderline] 0.1 ‘ns’ [non significant] 1

**Table 4:** All-cause and cause-specific premature mortality by region: low and mid versus high education level Rate Differences and Rate Ratio, females of Belgian origin aged 25–64 at census, Belgium, follow up 2001–11

			*Rate Ratio*									*Rate Ratio*								
	*Females*		Fla	Bxl	Wal	RD ratio Bxl - Fla	*p*	RD ratio Wal - Fla	*p*	RD ratio Wal -Bxl	*p*	Fla	Bxl	Wal	RR ratio Bxl- Fla	*p*	RR ratio Wal -Fla	*p*	RR ratio Wal -Bxl	*p*
*All-Cause*	*ALL CAUSES*	*Low*	107.4	196.2	163.5	1.83	***	1.52	***	0.83	*	1.6	1.8	1.8	1.12	**	1.07	**	0.96	ns
		*Mid*	52.2	79.9	86.0	1.53	*	1.65	***	1.08	ns	1.3	1.3	1.4	1.02	ns	1.07	*	1.04	ns
	*Avoidable*	*Low*	76.4	135.0	108.3	1.77	***	1.42	***	0.80	*	1.7	1.9	1.8	1.09	bl	1.04	ns	0.96	ns
		*Mid*	35.7	49.4	56.3	1.38	ns	1.58	***	1.14	ns	1.3	1.3	1.4	0.99	ns	1.06	ns	1.07	ns
	*Not Avoidable*	*Low*	31.0	61.3	55.2	1.97	***	1.78	***	0.90	ns	1.5	1.8	1.7	1.18	*	1.13	**	0.96	ns
		*Mid*	16.6	30.5	29.7	1.84	bl	1.79	**	0.97	ns	1.3	1.4	1.4	1.09	ns	1.09	bl	0.99	ns
*Broad Classes*	*ALL CANCERS*	*Low*	30.3	56.3	39.1	1.86	**	1.29	*	0.69	*	1.3	1.5	1.4	1.12	*	1.05	ns	0.93	ns
		*Mid*	17.5	21.5	22.5	1.23	ns	1.28	ns	1.05	ns	1.2	1.2	1.2	1.00	ns	1.03	ns	1.03	ns
	*ALL CIRCULAT. DIS.*	*Low*	30.8	53.8	39.6	1.74	***	1.29	***	0.74	**	2.7	2.9	2.5	1.07	ns	0.93	ns	0.87	ns
		*Mid*	16.9	20.2	21.3	1.20	ns	1.26	bl	1.05	ns	1.9	1.7	1.8	0.89	ns	0.94	ns	1.06	ns
	*OTH.NAT.DEATHS*	*Low*	34.7	70.6	67.5	2.04	***	1.95	***	0.96	ns	2.1	2.3	2.2	1.09	ns	1.06	ns	0.97	ns
		*Mid*	13.8	32.0	34.2	2.32	**	2.48	***	1.07	ns	1.4	1.6	1.6	1.10	ns	1.13	bl	1.02	ns
	*EXTERNAL CAUSES*	*Low*	11.6	15.5	17.3	1.34	ns	1.50	bl	1.12	ns	1.6	1.5	1.6	0.96	ns	1.01	ns	1.06	ns
		*Mid*	4.1	6.2	7.9	1.52	ns	1.95	ns	1.28	ns	1.2	1.2	1.3	1.00	ns	1.06	ns	1.06	ns
*Detailed*	*Lung Ca*	*Low*	12.5	28.3	15.9	2.27	***	1.28	*	0.56	**	2.1	2.3	1.9	1.11	ns	0.90	ns	0.81	ns
		*Mid*	6.0	9.7	6.1	1.60	ns	1.00	ns	0.63	ns	1.5	1.5	1.3	0.95	ns	0.87	ns	0.92	ns
	*Colorectal Ca*	*Low*	1.9	0.2	1.5	0.12	ns	0.75	ns	6.10	ns	1.2	1.0	1.2	0.83	ns	0.95	ns	1.14	ns
		*Mid*	0.5	1.1	0.4	2.15	ns	0.81	ns	0.38	ns	1.1	1.1	1.1	1.03	ns	0.99	ns	0.96	ns
	*Lip-Or.Cav-Phar.Ca*	*Low*	0.6	3.1	1.2	5.27	**	2.00	ns	0.38	bl	1.5	5.3	1.8	3.62	*	1.23	ns	0.34	bl
		*Mid*	0.1	2.6	1.0	47.08	*	18.02	bl	0.38	ns	1.0	4.6	1.7	4.37	*	1.60	ns	0.37	ns
	*Liver Cancer*	*Low*	0.9	0.8	1.6	0.92	ns	1.76	ns	1.91	ns	1.7	1.4	2.5	0.83	ns	1.49	ns	1.80	ns
		*Mid*	0.3	0.1	1.8	0.27	ns	6.45	*	24.05	ns	1.2	1.0	2.7	0.85	ns	2.23	*	2.61	bl
	*Breast Ca*	*Low*	1.7	2.7	1.8	1.60	ns	1.09	ns	0.68	ns	1.1	1.1	1.1	1.02	ns	1.01	ns	0.99	ns
		*Mid*	1.7	−2.1	0.0	−1.23	ns	−0.01	ns	0.01	ns	1.1	0.9	1.0	0.89	ns	0.95	ns	1.06	ns
	*Isc.Heart Dis.*	*Low*	10.8	17.0	15.3	1.57	*	1.42	***	0.90	ns	3.5	3.0	3.2	0.87	ns	0.93	ns	1.07	ns
		*Mid*	5.4	4.1	7.0	0.77	ns	1.30	ns	1.70	ns	2.2	1.5	2.0	0.67	bl	0.90	ns	1.35	ns
	*Cer.vasc.Dis/HTA*	*Low*	8.3	13.0	9.0	1.56	bl	1.08	ns	0.69	ns	2.3	2.5	2.1	1.06	ns	0.90	ns	0.85	ns
		*Mid*	5.2	4.2	6.2	0.80	ns	1.18	ns	1.47	ns	1.8	1.5	1.8	0.80	ns	0.95	ns	1.19	ns
	*C.o.p.d.*	*Low*	5.6	10.5	12.4	1.89	**	2.22	***	1.17	ns	4.6	4.1	4.1	0.89	ns	0.89	ns	1.00	ns
		*Mid*	2.6	4.2	5.5	1.64	ns	2.14	**	1.30	ns	2.7	2.3	2.4	0.84	ns	0.90	ns	1.06	ns
	*Alc. Rel Dth*	*Low*	2.5	10.4	10.6	4.24	**	4.33	***	1.02	ns	1.4	2.2	2.0	1.53	*	1.38	**	0.91	ns
		*Mid*	2.0	8.4	6.9	4.21	*	3.46	***	0.82	ns	1.4	2.0	1.6	1.45	bl	1.21	ns	0.84	ns
	*Diabetes*	*Low*	2.1	3.2	2.9	1.53	ns	1.38	ns	0.90	ns	3.7	2.0	2.3	0.56	ns	0.62	ns	1.12	ns
		*Mid*	0.8	−0.8	0.8	−1.01	ns	0.98	ns	−0.97	ns	2.1	0.7	1.4	0.36	bl	0.66	ns	1.85	ns
	*Mental/neurol. Dis*	*Low*	5.0	7.5	10.6	1.49	ns	2.10	***	1.41	ns	1.7	2.0	2.0	1.23	ns	1.23	ns	1.00	ns
		*Mid*	1.8	5.9	4.9	3.25	bl	2.70	*	0.83	ns	1.2	1.8	1.5	1.46	ns	1.19	ns	0.82	ns
	*Ill Defined*	*Low*	0.8	4.5	4.8	5.64	*	6.01	***	1.06	ns	1.2	1.8	1.9	1.43	ns	1.55	*	1.08	ns
		*Mid*	−0.2	2.1	2.6	−8.94	ns	−11.09	**	1.24	ns	0.9	1.4	1.5	1.47	ns	1.62	*	1.10	ns
	*Suicide*	*Low*	4.4	4.3	4.9	0.97	ns	1.11	ns	1.14	ns	1.4	1.2	1.3	0.90	ns	0.97	ns	1.08	ns
		*Mid*	1.5	3.5	1.5	2.41	ns	1.00	ns	0.41	ns	1.1	1.2	1.1	1.05	ns	0.98	ns	0.93	ns
	*Transport Acc.*	*Low*	3.4	2.8	5.7	0.83	ns	1.66	ns	1.99	ns	2.1	2.1	2.1	0.97	ns	0.96	ns	0.99	ns
		*Mid*	0.9	−1.5	2.1	−1.72	*	2.43	ns	−1.41	*	1.3	0.4	1.4	0.34	bl	1.08	ns	3.13	bl

Significance codes: 0 ‘***’ 0.001 ‘**’ 0.01 ‘*’ 0.05 ‘bl’ [borderline] 0.1 ‘ns’ [non significant] 1

In both sexes, the RDs differed considerably between regions. The highest RDs were observed in Brussels, followed by Wallonia and Flanders. All-cause mortality RDs were equal to 464, 395 and 252 in males and 196, 164 and 107 in females, in Brussels, Wallonia and Flanders, respectively.

The ratios between the region-specific educational RDs for all-cause mortality were almost identical for men and women: 1.84, 1.57 and 0.85 in men and 1.83, 1.52 and 0.83 in women for Brussels-Flanders, Wallonia-Flanders and Wallonia-Brussels respectively.

In cause-specific mortality, educational RDs were most pronounced in Brussels as well. Particularly, the Brussels-Flanders ratios were elevated with RDs higher than 3 for alcohol-related deaths, liver cancer, diabetes, mental and neurological diseases in men. For most of these causes, the mid versus high Brussels-Flanders RD ratios were very high too.

The Wallonia-Flanders RDs ratios were slightly smaller than the Brussels-Flanders RDs ratios; and most of the Brussels-Wallonia RDs ratios were not statistically significant.

A notable exception to this general picture was the RDs of transport accidents mortality, a very rare cause of death in Brussels. For prostate cancer, the Brussels versus Flanders RD ratio was reversed but not significant. Furthermore the RDs were slightly higher in Wallonia than in Brussels for COPD. In women, the cause-specific regional differences in RDs exceeded 3 also for alcohol-related deaths (Brussels versus Flanders and Wallonia versus Flanders) and lip-oral cavity-pharynx cancers.

Relative inequalities (RRs): the ratios between region-specific educational RRs (Tables 3 and 4, right part) were smaller than the above described ratios between educational RDs: for the all-cause mortality and for Brussels-Flanders, Wallonia-Flanders and Wallonia-Brussels they are respectively equal to 1.15, 1.04 and 0.91 in men, and 1.12, 1.07 and 0.96 in women. The Brussels-Flanders RR ratios in men were higher for cancers (highest ratios observed for lip-oral cavity and pharynx and liver cancers) and circulatory diseases than for other natural deaths, with however high RRs ratios for diabetes, mental and neurological diseases and alcohol-related deaths. The Wallonia-Flanders ratios were more moderate, exceeding 1.3 only for COPD. In women, most cause-specific RR-ratios comparing regions were not significant.

#### Population attributable fractions (PAF) and their decomposition into specific causes by region

In males, the fraction of all deaths that would have been avoided if the mortality of the total population were equal to the one observed in the highest EL (the total PAF) was respectively 13% and 11% higher in Brussels and Wallonia than in Flanders. In females, the PAFs of Brussels and Wallonia exceeded the one of Flanders with respectively 14 and 20% (Table 5).

**Table 5:** Decomposition of the total population attributable fraction in specific causes of death by region in Belgians aged 25–64 at census 2001, 10 years follow up

		*PAF, Fla*	*PAF, Bxl*	*PAF, Wal*	*Bxl* vs *Fla*	*p*	*Wal* vs *Fla*	*p*	*Wal* vs *Bxl*	*p*
*MALES*										
* All-Cause*	*ALL CAUSES*	37.6%	42.4%	41.7%	1.13	*	1.11	***	0.98	ns
	*Avoidable*	28.9%	30.5%	30.0%	1.05	ns	1.04	ns	0.99	ns
	*Not Avoidable*	8.7%	12.0%	11.6%	1.37	**	1.33	***	0.97	ns
* Broad Classes*	*ALL CANCERS*	12.3%	12.4%	11.0%	1.01	ns	0.90	bl	0.89	ns
	*ALL CIRCULAT. DIS.*	9.1%	9.9%	9.3%	1.09	ns	1.02	ns	0.94	ns
	*OTH.NAT.DEATHS*	9.4%	14.7%	14.7%	1.56	***	1.56	***	1.00	ns
	*EXTERNAL CAUSES*	6.9%	5.4%	6.7%	0.79	bl	0.97	ns	1.23	ns
* Detailed*	*Lung Ca*	7.0%	5.3%	6.0%	0.76	**	0.85	**	1.12	ns
	*Colorectal Ca*	0.5%	0.9%	0.3%	1.59	ns	0.54	ns	0.34	bl
	*Lip-Or.Cav-Phar.Ca*	0.8%	1.1%	0.8%	1.39	ns	0.95	ns	0.69	ns
	*LiverCancer*	0.2%	0.4%	0.3%	2.02	ns	1.20	ns	0.59	ns
	*Prostate Ca*	0.2%	0.0%	0.2%	0.10	ns	1.26	ns	12.11	ns
	*Isc.Heart Dis.*	4.5%	4.6%	4.6%	1.01	ns	1.02	ns	1.01	ns
	*Cer.vasc.Dis/HTA*	1.4%	1.7%	1.6%	1.21	ns	1.11	ns	0.92	ns
	*C.o.p.d.*	2.0%	2.6%	2.8%	1.26	bl	1.38	***	1.09	ns
	*Alc. Rel Dth*	1.4%	3.0%	2.6%	2.18	***	1.89	***	0.87	ns
	*Diabetes*	0.4%	0.8%	0.8%	1.73	bl	1.70	**	0.98	ns
	*Mental/neurol. Dis*	1.4%	2.5%	2.6%	1.81	**	1.82	***	1.01	ns
	*Ill Defined*	0.7%	1.4%	1.5%	1.95	bl	2.06	***	1.06	ns
	*Suicide*	3.4%	2.8%	3.1%	0.83	ns	0.91	ns	1.10	ns
	*Transport Acc.*	1.8%	0.6%	1.6%	0.36	***	0.94	ns	2.61	**
*FEMALES*										
* All-Cause*	*ALL CAUSES*	29.5%	33.6%	35.4%	1.14	ns	1.20	***	1.05	ns
	*Avoidable*	20.2%	22.1%	22.9%	1.09	ns	1.13	bl	1.04	ns
	*Not Avoidable*	9.3%	11.6%	12.5%	1.24	ns	1.35	**	1.08	ns
* Broad Classes*	*ALL CANCERS*	8.6%	8.7%	8.5%	1.01	ns	0.99	ns	0.98	ns
	*ALL CIRCULAT. DIS.*	9.4%	9.0%	8.8%	0.95	ns	0.94	ns	0.99	ns
	*OTH.NAT.DEATHS*	9.5%	13.6%	15.3%	1.44	**	1.61	***	1.12	ns
	*EXTERNAL CAUSES*	2.1%	2.4%	2.8%	1.15	ns	1.36	ns	1.18	ns
* Detailed*	*Lung Ca*	3.4%	4.1%	3.2%	1.20	ns	0.93	ns	0.77	ns
	*Colorectal Ca*	0.5%	−0.1%	0.3%	−0.13	ns	0.55	ns	−4.18	ns
	*Lip-Or.Cav-Phar.Ca*	0.1%	0.5%	0.3%	3.73	*	2.15	ns	0.58	ns
	*LiverCancer*	0.3%	0.1%	0.4%	0.41	ns	1.45	ns	3.52	ns
	*Breast Ca*	0.4%	0.4%	0.3%	0.89	ns	0.76	ns	0.85	ns
	*Isc.Heart Dis.*	3.4%	2.7%	3.3%	0.79	ns	0.96	ns	1.21	ns
	*Cer.vasc.Dis/HTA*	2.4%	2.1%	2.1%	0.89	ns	0.86	ns	0.96	ns
	*C.o.p.d.*	1.8%	2.0%	2.7%	1.10	ns	1.49	***	1.36	bl
	*Alc. Rel Dth*	0.8%	2.2%	2.2%	2.93	**	2.94	***	1.00	ns
	*Diabetes*	0.6%	0.4%	0.6%	0.70	ns	1.06	ns	1.51	ns
	*Mental/neurol. Dis*	1.2%	2.0%	2.3%	1.66	ns	1.91	**	1.15	ns
	*Ill Defined*	0.2%	0.9%	1.2%	4.26	ns	5.77	***	1.35	ns
	*Suicide*	0.6%	0.9%	0.7%	1.33	ns	1.02	ns	0.76	ns
	*Transport Acc.*	0.5%	0.0%	0.7%	0.06	ns	1.37	ns	21.44	bl

Significance codes: 0 ‘***’ 0.001 ‘**’ 0.01 ‘*’ 0.05 ‘bl’ [borderline] 0.1 ‘ns’ [non significant] 1

The analysis of the cause-specific contribution to the PAF by region (Table 5), revealed that among men the contribution of lung cancer to educational inequalities was higher in Flanders (7.0%) than in the other regions (5.3% and 6.0% respectively in Brussels and Wallonia), while that of COPD, alcohol-related deaths, diabetes and mental/neurological diseases was higher in Wallonia and Brussels than in Flanders.

In women, the specific contribution to the PAFs of COPD, alcohol-related deaths and mental-neurological diseases was higher in Wallonia than in Flanders, while that of alcohol-related mortality and lip, oral cavity and pharynx cancers were higher in Brussels than in Flanders.

### Ranking of the causes of death based on their impact on inequalities

**Fig. 2:**
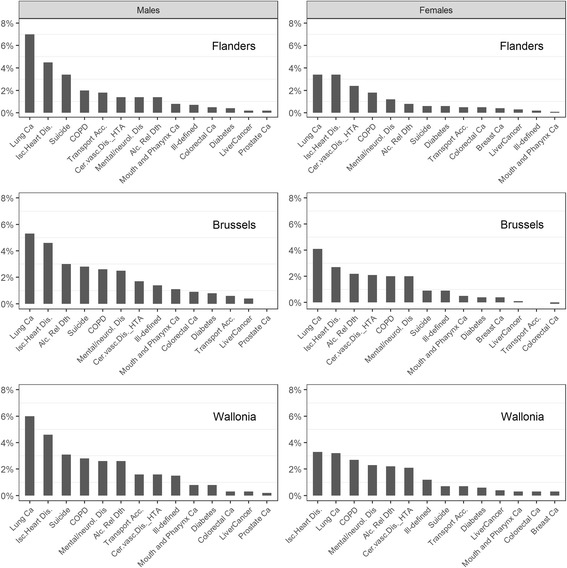
Cause-specific population attributable fractions by gender and region. People of Belgian origin aged 25–64 at census, Belgium, follow up 2001–11

The ranking of the specific contribution of each detailed cause, by sex and region, is shown in Fig. 2. In men, the ranking of the main contributors was quite similar between regions, with lung cancers and ischemic heart diseases at the top. Alcohol-related deaths and mental/neurological diseases contributed more to the PAFs in Wallonia and Brussels than in Flanders, while transport accidents contributed much less to the PAF in Brussels than in the other regions.

In women, ischemic heart diseases ranked first in Wallonia, followed by lung cancer. In Flanders, they ranked equally. In Brussels, lung cancer ranked as first contributor to the PAF. Colorectal cancer ranked lower in Wallonia and Brussels than in Flanders.

## Discussion

During the 2000s, Belgium was characterized by a high premature mortality rate compared to the average of the EU15 countries [17]. As overall country-level rates can hide important disparities, this paper examined the regional and educational health gaps in premature mortality rates, compared the educational inequalities across the Belgian regions and decomposed the inequality population-level impact into its main causes. The study focused on people of Belgian nationality.

### Summary of previous work

Previous research has documented important mortality differences at the regional or district level in Belgium [10–17] with consistently higher rates in Wallonia (especially in the poorest districts of the Hainaut province) and Brussels as compared to Flanders (with the lowest rates in the eastern districts of the province of Limburg) since World War 2. Until the turn of the century, the link between individual SE characteristics and mortality could only be investigated through ecological studies because of the lack of appropriate data at the individual level. The constitution, in the early 2000s, of a “National Mortality Database” [6, 20], aiming to perform a population-based mortality follow-up, finally allowed for the study of SE mortality differentials in Belgium. Several studies, first based on the 1991 census and later on the 2001 census, assessed the magnitude of the SE health gap (and its change over time) in terms of differences in life expectancy [5], health expectancy [7, 8], all-cause and cause-specific mortality [6, 9] in Belgium.

Results from individual studies about inequalities are difficult to compare with each other because they generally present variations in the design of the follow up, the age limits and the standard population. Moreover, by focusing on the Belgian population, our results are not comparable with studies that also included migrant populations. However, a recent European study included Belgium in cross-country comparisons of inequalities in mortality [1, 2]. This study used 2 years of follow up and focused on people aged 30–74 at entry, which is 10 years older than in our study. Major inequalities were revealed in the Eastern European countries (with RDs in men exceeding 1500 per 100,000 PY and RRs situated between 2.6 and 3.3), rather large inequalities in Northern Europe (with RDs in men between 500 and 700 per 100,000 PY and RRs around 2), an inhomogeneous pattern in Western Europe (RDs in men between 300 and 600 per 100,000 PY, RRs between 1.6 and 2.4), and lower inequalities in Southern Europe (RDs in men between 250 and 400 per 100,000 PY and RRs around 1.6). As compared to the other European countries, inequalities in Belgium could be qualified as moderately high, with a RD and RR in men respectively equal to 385 per 100,000 PY and 1.86, while the mean RD for all the countries was 636 (range: 234–1696) and the mean RR was 2.1 (range: 1.51–3.26).

Only few studies jointly analyzed the effects of place of residence (region, province, district) and SE characteristics. Deboosere et al. [17] examined the district-level patterns of all-cause mortality in the 1990s with and without adjustment for individual SE variables and concluded that individual SE characteristics accounted for half of the mortality risk excess in the poorer districts of the old industrial belt of the Walloon region. Van Hemelrijck et al. [19] found a weak effect of area-level unemployment and percentage of laborers on the sub-district mortality RR, in addition to the effect of individual SE characteristics.

The analysis of inequalities by region has however not yet been performed by specific cause of death.

### Summary of main findings

Large premature mortality excesses were observed in Brussels and Wallonia as compared to Flanders, which is in line with previous geographical mortality studies in Belgium. Our focus on people of Belgian origin increased the magnitude of those mortality excesses even further compared to studies including all residents, which is expected given both the mortality advantage in first generation migrants [21, 22, 42] and their higher representation in Wallonia and particularly in Brussels. In line with previous findings of Deboosere et al. in the 1990s [18], our analysis showed significant regional differences for premature mortality at the global level and within each level of education. The results of the Poisson regression also revealed that the educational distributions do not explain at all the regional differences in mortality. The level of employment in men accounts for a small part of the regional differences, while up to half of the differences can be explained by the housing score, which is a proxy for wealth.

With respect to inequalities in all-cause mortality, the largest inequalities were observed in Brussels and the lowest in Flanders, independently of the three inequality indices used. Important regional differences in absolute inequalities (RDs) were observed, but relative differences (RRs and PAFs) were weak. This pattern was observed for all-cause as well as for most specific causes of premature mortality.

The ranking of the cause-specific PAFs revealed a higher health impact of inequalities in causes combining a high mortality rate and a high relative inequality, with lung cancer and ischemic heart disease on top for all regions and both sexes. Suicide, COPD and cerebrovascular diseases also ranked high. The ranking of the contribution of the specific causes of death showed few differences between the regions.

### Interpretation and policy implication

Our results clearly show that the mortality excess in Brussels and Wallonia is still persisting. While this health gap appears to be difficult to eliminate in the context of unequal economical background between the different regions, this study, as well as two previous ones, showed that individual SE variables accounted only for half of the regional differences. The distribution of poverty in the regions (approached by the housing score), and to a lower extent, the employment status (in men only) are the main individual SE factors that explaining half of the regional difference, while educational level does not account. Up to now, the residual regional effect on mortality has not yet been elucidated, and could involve several factors such as: other macroeconomic variables that could not be captured by the existing data, cultural habits leading to less healthy lifestyle in some regions, effects of indoor and outdoor pollution and/or differences in health policies or health care management. In a multilevel analysis, Van Hemelrijck et al. [19] showed small additional effects of two aggregated variables, the level of unemployment and the percentage of laborers in the district. This means that an important unexplained residual regional effect remains after adjusting for individual and some macro SE variables. Further studies should try to disentangle the respective roles of those other risk factors in order to support policies oriented to reducing the health gap.

Larger inequalities were observed in the regions that also had higher mortality rates, and particularly in Brussels. The magnitude of the regional differences in inequality differs when it is expressed in absolute (RDs) or relative (RR and PAFs) terms, which is quite expected since the ASMR varies greatly between the regions. Indeed, when a region has high mortality rates, larger RDs will be observed for a same RR between ELs. In this study, however, we also observed higher relative inequalities in the regions with higher mortality rates, which is less expected. Indeed, since high RRs are more easily observed when the denominator is small (low mortality rates), observing a higher RR in a region that also have high rates, as compared to a region with lower rates, can only be observed when inequalities are strong.

The more unfavorable situation of Belgians living in Brussels, with respect to both mortality rates and inequality as compared to Flanders and even to Wallonia, should be interpreted in the light of the particular situation of this region. Indeed, in contrast to the other regions, the Brussels region is actually a big city without rural/suburban areas. There is a well-known ‘town attraction’, where people with social problems tend to move towards big cities to search for solutions, leading to some poverty concentration.

The PAF decomposition in specific causes is of major interest for policy-making since it can help set priorities by addressing inequalities in the causes with the highest population level impact. Causes of death combining high mortality rates and high inequalities rank highest. It is important to note that the causes ranking highest for the population-level inequality impact are related to major risk factors and in some measure amenable to health promotion measures. The ranking varies little by region, with the exception of a higher ranking of the PAF for alcohol-related deaths in Brussels, together with a lower PAF for transport accidents, which is expected given its lower mortality rate. Policies addressing inequalities in smoking, obesity and cholesterol level, as well as the medical management of ischemic and cerebrovascular diseases can be recommended in all three regions; ideally, a comprehensive “national and regional” policy should be implemented. Particular attention should be paid to the health of the low educated people in Brussels.

### Strengths and limitations

For the first time, inequalities were comprehensively measured and compared at the regional level. In the Belgian situation, where many public health responsibilities are transferred to the regional level, the calculation of inequality indices for each region is highly relevant to support regional policies. Inequalities were analyzed both for all-cause and for cause-specific premature mortality, using various inequality indices, namely the absolute and relative RDs and the PAF. By taking into account the EL distribution, the PAF is probably the best population-based measure to estimate the impact of inequalities on the total population health. It is also the first time that the cause-specific mortality inequalities were expressed by region in terms of a contribution to the total PAF.

By combining national census data with mortality data, the study covered practically the complete Belgian population. The large size of the cohort ensures a good statistical power.

It is important to note that the conclusions only apply to the Belgian population. Migrants registered with another nationality at birth were not included. As they represented a substantial fraction of the population, especially in Brussels, and they also experience lower mortality rates than the Belgians, our findings should not be extrapolated to the whole population. This choice on focusing on Belgian people only was made in first instance to keep a certain homogeneity in the population under study, since migrants have various backgrounds and socio-economic levels. Secondly, as the proportion of migrants varies between regions, nationality may represent a confounding factor in regional comparisons. Health inequalities should also be specifically studied in migrants, taking into account their ethnic specificities.

By definition, people living in Belgium without being registered were also not part of the studied population.

The percentage of missing values for the EL indicator was rather low, with some disparities between regions. We did not impute the missing values as we cannot expect them to be missing at random. Instead, we just treated them as a separate category. Since the study population included people of Belgian origin only, the language was probably not expected to be a barrier to answer the census questionnaire (as it could be in migrants). People hospitalized or severely sick at the time of the census were very likely not to have answered the questionnaire. Also part of the homeless people could not receive the post-mailed questionnaire; this led to missing values for all SE variables for the more sick or the more deprived people. In the Poisson models estimating RRs for all-cause mortality, the cases with missing SES were treated as a separate category for all three SE variables; as expected, those cases appeared to experience higher mortality rates than all other categories. This poorer health status in non-respondents is in line with previous findings related to the 1991 census [5]. In the subsequent analysis, we used as pairwise measures the low-versus-high and mid-versus-high RR and RD, and did not make use of the missing-value group; this is likely to lead to an underestimation of the inequalities (conservative bias).

Some inaccuracy in cause of deaths codification exists, resulting in 8% of ill-defined codes in the considered age group [17], and in an underestimation of cause specific rates. This could possibly be more the case in lower SE classes (if less efforts are made to get an accurate diagnosis in less than in more educated people), resulting in underestimated cause-specific inequalities (conservative effect).

Age limits for ‘premature mortality’ vary in literature. Our study covered people aged 25–64 at baseline, allowing them to reach at maximum 69 years at the end of the follow-up period. Our findings cannot be generalized to other age groups, and any comparison with other studies has to take the age range in consideration.

We chose the old European population as a standard population- even if a new one is now proposed by Eurostat - since this had been used in 2014 and 2016 European comparisons [43, 44]. Of course, the standardized rates are sensitive to the standard population, but most probably the impact on the inequalities will not be very important.

## Conclusions

Regional disparities in premature mortality persist in Belgium, with much higher rates in Brussels and Wallonia than in Flanders, and this within each EL. Individual SE characteristics only account for half of these regional differences, especially the unequal distribution of poverty and employment status between the regions. Explanations for the residual regional effect should be searched in macroeconomic characteristics, differences in lifestyles and in inside/outside air pollution, differences in health policies and in health care management.

Regional educational inequalities in premature mortality were studied for the first time and revealed higher absolute inequalities in Brussels and Wallonia compared to Flanders, as well as a weak excess in relative inequalities.

The PAF decomposition in specific causes and its ranking according to the highest population-level impact of the inequalities in mortality is important for the policy-making process, since it can help set priorities by addressing inequalities in the causes with the highest population level impact. Causes ranking highest for the population-level inequality impact are lung cancer, ischemic heart disease, suicide, cerebrovascular disease, most of which are related to major risk factors like alcohol or tobacco consumption, and in some extent amenable to health promotion measures. The rankings varied little by region, with the exception of a higher ranking of the PAF for alcohol-related deaths in Brussels, together with a lower PAF for transport accidents.

## Additional files

Additional file 1: Table S1a. Age-adjusted all-cause premature mortality rates (ASMR) in males by educational level, region and origin, Belgium 2000s; people aged 25–64 at census, 10 years follow up. Table S1b. Age-adjusted premature mortality rates (ASMR) in females by educational level, region and origin, Belgium 2000s; people aged 25–64 at census, 10 years follow up. Table S2 Codes of deaths selected for the analysis, in ICD9 and ICD10. Table S3a. Age-adjusted cause-specific premature mortality rate (ASMR), by region and educational level in Males, Belgium 2000s; Belgian men aged 25–64 at census, 10 years follow up. Table S3b. Age-adjusted cause-specific premature mortality rate (ASMR), by region and educational level in females, Belgium 2000s; Belgian women aged 25–64 at census, 10 years follow up. (PDF 605 kb)
