# Supplementary material for: Educational inequalities in premature mortality by region in the Belgian population in the 2000s
Source: Arch Public Health. 2017 Oct 16;75:44. doi: 10.1186/s13690-017-0212-x (PMC5641991; doi:10.1186/s13690-017-0212-x)
Supplement: Supplementary file 1 — Age-adjusted all-cause premature mortality rates (ASMR) in males by educational level, region and origin, Belgium 2000s; people aged 25–64 at census, 10 years follow up. Table S1b. Age-adjusted premature mortality rates (ASMR) in females by educational level, region and origin, Belgium 2000s; people aged 25–64 at census, 10 years follow up. Table S2 Codes of deaths selected for the analysis, in ICD9 and ICD10. Table S3a. Age-adjusted cause-specific premature mortality rate (ASMR), by region and educational level in Males, Belgium 2000s; Belgian men aged 25–64 at census, 10 years follow up. Table S3b. Age-adjusted cause-specific premature mortality rate (ASMR), by region and educational level in females, Belgium 2000s; Belgian women aged 25–64 at census, 10 years follow up. (PDF 605 kb) [file 13690_2017_212_MOESM1_ESM.pdf]

Educational inequalities in premature mortality by region in the Belgian population in the 2000s

ONLINE APPENDIX

Table of Contents

Appendix Table 1a. Age-adjusted all-cause premature mortality rates (ASMR) in males by educational level, region and origin, Belgium 2000s; people aged 25-64 at census, 10 years follow up. 2

Appendix Table 1b. Age-adjusted premature mortality rates (ASMR) in females by educational level, region and origin, Belgium 2000s; people aged 25-64 at census, 10 years follow up. 3

Appendix Table 2 Codes of deaths selected for the analysis, in ICD9 and ICD10. 4

Appendix Table 3a. Age-adjusted cause-specific premature mortality rate (ASMR), by region and educational level in Males, Belgium 2000s; Belgian men aged 25-64 at census, 10 years follow up. 5

Appendix Table3b. Age-adjusted cause-specific premature mortality rate (ASMR), by region and educational level in females, Belgium 2000s; Belgian women aged 25-64 at census, 10 years follow up. 6

Appendix Table 1a. Age-adjusted all-cause premature mortality rates (ASMR) in males by educational level, region and origin, Belgium 2000s; people aged 25-64 at census, 10 years follow up.

		ASMR BELGIUM	*ASMR Fla*	*ASMR Bxl*	*ASMR Wal*
All Residents	*ALL EL*	**506.5**	432.0	565.1	635.5
	Low EL	**593.4**	513.1	661.3	729.3
	Mid EL	**445.3**	385.0	534.3	559.9
	High EL	**312.1**	271.3	351.2	379.0
	Missing	**892.3**	783.0	817.4	1071.9
Belgian	*ALL EL*	**514.2**	433.6	659.4	665.7
	Low EL	**614.7**	522.4	843.9	783.0
	Mid EL	**450.1**	386.5	600.8	582.0
	High EL	**313.9**	270.4	379.7	388.3
	Missing	**1003.6**	827.8	1223.4	1201.0
Migrants	*ALL EL*	**463.1**	417.1	433.3	514.8
	Low EL	**501.4**	451.4	470.9	550.8
	Mid EL	**412.7**	367.0	403.7	458.0
	High EL	**297.0**	284.6	286.7	315.7
	Missing	**632.9**	600.3	542.6	746.1

Note: Rates are expressed per 100,000 person-years and age standardized using the European population. Migrants are people from foreign origin officially registered as residents in Belgium.

Appendix Table 1b. Age-adjusted premature mortality rates (ASMR) in females by educational level, region and origin, Belgium 2000s; people aged 25-64 at census, 10 years follow up.

		ASMR BELGIUM	*ASMR Fla*	*ASMR Bxl*	*ASMR Wal*
All Residents	*ALL EL*	**271.0**	238.4	311.3	320.6
	Low EL	**304.2**	271.1	343.5	355.6
	Mid EL	**246.0**	219.8	298.9	288.3
	High EL	**189.9**	168.5	219.3	214.6
	Missing	**468.5**	412.7	450.9	563.1
Belgian	*ALL EL*	**273.3**	237.8	351.6	332.8
	Low EL	**314.0**	275.0	429.6	378.4
	Mid EL	**248.0**	219.8	313.2	300.9
	High EL	**189.6**	167.6	233.3	214.9
	Missing	**534.8**	439.8	689.9	641.3
Migrants	*ALL EL*	**257.7**	251.1	245.5	268.9
	Low EL	**268.6**	267.5	250.7	279.3
	Mid EL	**230.6**	221.3	259.0	224.4
	High EL	**194.1**	185.9	177.6	216.7
	Missing	**339.9**	334.7	296.1	386.0

Note: Rates are expressed per 100,000 person-years and age standardized using the European population. Migrants are people from foreign origin officially registered as residents in Belgium.

Appendix Table 2 Codes of deaths selected for the analysis, in ICD9 and ICD10.

**Causes of deaths**	**ICD9**	**ICD10**
Circulatory Diseases	390-459	I00-I99
Cancers	140-209	C00-C99
Other natural causes	Rest(001-799)	Rest(A00-R99)
External causes	E800-E999	V01-Y98
Selected Avoidable Causes		
Lip-OralCav-Phar Ca	140-149	C00-C14
Colorectal Ca	153:154	C18-C21
LiverCancer	155	C22
Lung Ca	162	C33-C34
Breast Ca (women)	174	C50
Isc.Heart Dis.	410:414	I20:I25
Cer.vasc.Dis/HTA	401:405; 430:438	I10-I15; I60-I69
COPD	490:492,494,496	J40:J44, J47
Alc. Rel Dth	291, 303, 3050, 4255, 5353, 5710:5713, 5715, 5770, 5771	F10, K70, K73, K74, G312, G621, I426, K292, K852, K860
Diabetes	250	E10-E14
Suicide	950:959	X60-X84,Y870
Land Transport Acc.	800:829	V01:V89, Y85

Appendix Table 3a. Age-adjusted cause-specific premature mortality rate (ASMR), by region and educational level in Males, Belgium 2000s; Belgian men aged 25-64 at census, 10 years follow up.

		Low EL									Mid EL									High EL									ALL levels								
		ASMR Fla	ASMR Bxl	ASMR Wal	RR Bxl-Fl	p	RR Wal_Fl	p	RR Wal_Bx	p	ASMR Fla	ASMR Bxl	ASMR Wal	RR Bxl-Fl	p	RR Wal_Fl	p	RR Wal_Bx	p	ASMR Fla	ASMR Bxl	ASMR Wal	RR Bxl-Fl	p	RR Wal_Fl	p	RR Wal_Bx	p	ASMR Fla	ASMR Bxl	ASMR Wal	RR Bxl-Fl	p	RR Wal_Fl	p	RR Wal_Bx	p
All-Cause	ALL CAUSES	522.4	844	783	1.6	***	1.5	***	0.9	***	387	600.8	582	1.6	***	1.5	***	1	ns	270.4	379.7	388.3	1.4	***	1.4	***	1	ns	433.6	659.4	665.7	1.5	***	1.5	***	1	ns
	Avoidable	368.2	581	541.4	1.6	***	1.5	***	0.9	**	265	407.3	391.4	1.5	***	1.5	***	1	ns	173	240.1	251.8	1.4	***	1.5	***	1	ns	298.2	440.9	451.8	1.5	***	1.5	***	1	bl
	Not Avoidable	154.1	263	241.5	1.7	***	1.6	***	0.9	*	122	193.5	190.7	1.6	***	1.6	***	1	ns	97.4	139.6	136.4	1.4	***	1.4	***	1	ns	135.4	218.5	213.9	1.6	***	1.6	***	1	ns
Broad Classes	ALL CANCERS	194.7	276	247.2	1.4	***	1.3	***	0.9	***	152	204.6	186	1.3	***	1.2	***	0.9	bl	115.3	134.8	137.7	1.2	***	1.2	***	1	ns	168.6	216.6	211.2	1.3	***	1.3	***	1	ns
	ALL CIRCULAT. DIS.	114.5	182	161.5	1.6	***	1.4	***	0.9	**	84	125.2	118.8	1.5	***	1.4	***	0.9	ns	57.9	74.5	75.3	1.3	***	1.3	***	1	ns	97.3	140	137.3	1.4	***	1.4	***	1	ns
	OTH.NAT.DEATHS	119.7	261	244.2	2.2	***	2	***	0.9	bl	83.2	186.6	176.9	2.2	***	2.1	***	0.9	ns	59.4	113.2	112.1	1.9	***	1.9	***	1	ns	100	209.8	209.6	2.1	***	2.1	***	1	ns
	EXTERNAL CAUSES	93.4	126	130	1.3	***	1.4	***	1	ns	67.3	84.4	100.4	1.3	**	1.5	***	1.2	*	37.9	57.2	63.2	1.5	***	1.7	***	1.1	ns	67.6	93	107.6	1.4	***	1.6	***	1.2	***
Detailed	Lung Ca	75.3	99.9	96.1	1.3	***	1.3	***	1	ns	50.2	64.8	62.7	1.3	**	1.2	***	1	ns	30.6	37.8	37.3	1.2	*	1.2	***	1	ns	61	72.9	77	1.2	***	1.3	***	1.1	ns
	Colorectal Ca	15.9	22.2	15.7	1.4	**	1	ns	0.7	**	15.8	16.6	14.9	1.1	ns	0.9	ns	0.9	ns	12.8	12.5	13.6	1	ns	1.1	ns	1.1	ns	15.2	18.3	15.5	1.2	**	1	ns	0.9	*
	Lip-Or.Cav-Phar.Ca	9.9	18.3	13.8	1.8	***	1.4	***	0.8	*	7	12.1	9.5	1.7	*	1.4	**	0.8	ns	4.8	6.1	5.4	1.3	ns	1.1	ns	0.9	ns	8.4	13.6	10.7	1.6	***	1.3	***	0.8	**
	LiverCancer	4.5	9.4	8.2	2.1	***	1.8	***	0.9	ns	4.7	10.7	7.6	2.3	**	1.6	***	0.7	ns	3.5	5.6	5.9	1.6	bl	1.7	***	1.1	ns	4.4	8.4	7.6	1.9	***	1.7	***	0.9	ns
	Prostate Ca	6.3	5.1	7.8	0.8	ns	1.2	**	1.5	*	5.7	7.1	6.6	1.3	ns	1.2	ns	0.9	ns	5.3	6.4	5.4	1.2	ns	1	ns	0.8	ns	6	6.6	6.9	1.1	ns	1.1	*	1.1	ns
	Isc.Heart Dis.	54.7	86.2	80.1	1.6	***	1.5	***	0.9	ns	38.6	57.9	55.2	1.5	***	1.4	***	1	ns	25.8	35.7	34.7	1.4	***	1.3	***	1	ns	45.5	65.9	65.5	1.4	***	1.4	***	1	ns
	Cer.vasc.Dis/HTA	19	27.1	28.5	1.4	***	1.5	***	1.1	ns	13.8	24.2	21.8	1.8	**	1.6	***	0.9	ns	10.2	11.1	14.1	1.1	ns	1.4	***	1.3	bl	16.5	22.6	24.7	1.4	***	1.5	***	1.1	ns
	C.o.p.d.	17.3	33.4	34.4	1.9	***	2	***	1	ns	8	16.6	14.6	2.1	***	1.8	***	0.9	ns	4.5	7.4	6.7	1.6	*	1.5	**	0.9	ns	13.4	24.4	25.5	1.8	***	1.9	***	1	ns
	Alc. Rel Dth	19.5	55.1	48.3	2.8	***	2.5	***	0.9	bl	16.1	47.2	43.8	2.9	***	2.7	***	0.9	ns	11.4	23.5	27.2	2.1	***	2.4	***	1.2	ns	17.4	43.3	44.5	2.5	***	2.6	***	1	ns
	Diabetes	5.2	12.5	11.4	2.4	***	2.2	***	0.9	ns	3.4	5.2	8.9	1.5	ns	2.6	***	1.7	*	2.4	3.5	4.6	1.5	ns	1.9	***	1.3	ns	4.3	8.6	9.6	2	***	2.2	***	1.1	ns
	Mental/neurol. Dis	18.9	40.5	40.3	2.1	***	2.1	***	1	ns	13.2	29.9	29.8	2.3	***	2.3	***	1	ns	9.5	12	18.3	1.3	ns	1.9	***	1.5	***	15.6	28.7	35.3	1.8	***	2.3	***	1.2	***
	Ill Defined	10.7	29.2	24.5	2.7	***	2.3	***	0.8	ns	8.5	19.1	18.6	2.2	***	2.2	***	1	ns	6.4	13	11.7	2	***	1.8	***	0.9	ns	9.5	22.1	21.5	2.3	***	2.3	***	1	ns
	Suicide	50.4	69.3	64.1	1.4	**	1.3	***	0.9	ns	36.3	50.9	50.8	1.4	**	1.4	***	1	ns	21.7	34.5	32.2	1.6	***	1.5	***	0.9	ns	36.5	53.1	52.8	1.5	***	1.4	***	1	ns
	Transport Acc.	22	13.7	32.6	0.6	**	1.5	***	2.4	***	17	9.3	25.7	0.5	***	1.5	***	2.8	***	8	6.6	15.2	0.8	ns	1.9	***	2.3	***	15.6	10.7	26.1	0.7	***	1.7	***	2.4	***

Note: Rates are expressed per 100,000 person-years

Appendix Table3b. Age-adjusted cause-specific premature mortality rate (ASMR), by region and educational level in females, Belgium 2000s; Belgian women aged 25-64 at census, 10 years follow up.

		Low EL									Mid EL								
		ASMR Fla	ASMR Bxl	ASMR Wal	RR Bxl-Fl	p	RR Wal_Fl	p	RR Wal_Bx	p	ASMR Fla	ASMR Bxl	ASMR Wal	RR Bxl-Fl	p	RR Wal_Fl	p	RR Wal_Bx	p
All-Cause	ALL CAUSES	275	429.6	378.4	1.6	***	1.4	***	0.9	***	219.8	313.2	300.9	1.4	***	1.4	***	1	ns
	Avoidable	184.5	291.3	246.6	1.6	***	1.3	***	0.8	***	143.8	205.7	194.7	1.4	***	1.4	***	0.9	ns
	Not Avoidable	90.5	138.3	131.8	1.5	***	1.5	***	1	ns	76	107.5	106.2	1.4	***	1.4	***	1	ns
Broad Classes	ALL CANCERS	128.2	175.5	143.2	1.4	***	1.1	***	0.8	***	115.4	140.7	126.6	1.2	***	1.1	***	0.9	*
	ALL CIRCULAT. DIS.	49.1	82.4	65.9	1.7	***	1.3	***	0.8	***	35.1	48.8	47.5	1.4	***	1.4	***	1	ns
	OTH.NAT.DEATHS	65.9	125.2	122.3	1.9	***	1.9	***	1	ns	44.9	86.6	89.1	1.9	***	2	***	1	ns
	EXTERNAL CAUSES	31.8	46.4	47	1.5	***	1.5	***	1	ns	24.3	37.1	37.6	1.5	**	1.5	***	1	ns
Detailed	Lung Ca	23.7	49.5	33.8	2.1	***	1.4	***	0.7	***	17.3	30.9	24	1.8	***	1.4	***	0.8	*
	Colorectal Ca	10.4	12.2	10.4	1.2	ns	1	ns	0.8	ns	9	13.1	9.3	1.5	*	1	ns	0.7	bl
	Lip-Or.Cav-Phar.Ca	1.8	3.8	2.6	2.1	*	1.4	**	0.7	ns	1.3	3.3	2.4	2.5	*	1.9	**	0.7	ns
	LiverCancer	2.2	3	2.7	1.3	ns	1.2	ns	0.9	ns	1.6	2.2	2.9	1.4	ns	1.8	*	1.3	ns
	Breast Ca	34.3	38.9	32.1	1.1	ns	0.9	bl	0.8	*	34.4	34.1	30.2	1	ns	0.9	*	0.9	ns
	Isc.Heart Dis.	15.1	25.4	22.3	1.7	***	1.5	***	0.9	ns	9.7	12.5	13.9	1.3	ns	1.4	***	1.1	ns
	Cer.vasc.Dis/HTA	14.5	21.7	17.1	1.5	**	1.2	*	0.8	*	11.4	12.9	14.3	1.1	ns	1.2	*	1.1	ns
	C.o.p.d.	7.1	13.9	16.3	2	***	2.3	***	1.2	ns	4.1	7.6	9.5	1.8	*	2.3	***	1.2	ns
	Alc. Rel Dth	8	19.1	21.3	2.4	***	2.7	***	1.1	ns	7.6	17	17.6	2.3	***	2.3	***	1	ns
	Diabetes	2.9	6.3	5.1	2.2	**	1.8	***	0.8	ns	1.6	2.2	3	1.4	ns	1.9	**	1.4	ns
	Mental/neurol. Dis	12.9	14.9	20.9	1.2	ns	1.6	***	1.4	**	9.6	13.2	15.2	1.4	bl	1.6	***	1.2	ns
	Ill Defined	4.2	10.3	10	2.5	***	2.4	***	1	ns	3.1	8	7.9	2.5	**	2.5	***	1	ns
	Suicide	17	24.8	21	1.5	*	1.2	*	0.8	ns	14.1	24	17.5	1.7	**	1.2	**	0.7	bl
	Transport Acc.	6.4	5.5	11.1	0.9	ns	1.7	**	2	**	3.9	1.2	7.5	0.3	***	1.9	***	6.4	***

		High EL									ALL levels								
		ASMR Fla	ASMR Bxl	ASMR Wal	RR Bxl-Fl	p	RR Wal_Fl	p	RR Wal_Bx	p	ASMR Fla	ASMR Bxl	ASMR Wal	RR Bxl-Fl	p	RR Wal_Fl	p	RR Wal_Bx	p
All-Cause	ALL CAUSES	167.6	233.3	214.9	1.4	***	1.3	***	0.9	*	237.8	351.6	332.8	1.5	***	1.4	***	0.9	***
	Avoidable	108.1	156.3	138.3	1.4	***	1.3	***	0.9	**	156.2	233.9	214.5	1.5	***	1.4	***	0.9	***
	Not Avoidable	59.5	77	76.6	1.3	***	1.3	***	1	ns	81.6	117.7	118.3	1.4	***	1.5	***	1	ns
Broad Classes	ALL CANCERS	97.9	119.2	104.1	1.2	***	1.1	*	0.9	**	118.3	149.6	132.4	1.3	***	1.1	***	0.9	***
	ALL CIRCULAT. DIS.	18.2	28.6	26.2	1.6	***	1.4	***	0.9	ns	40.5	60.1	55.6	1.5	***	1.4	***	0.9	*
	OTH.NAT.DEATHS	31.2	54.6	54.9	1.8	***	1.8	***	1	ns	53.7	102.6	105.7	1.9	***	2	***	1	ns
	EXTERNAL CAUSES	20.3	30.9	29.7	1.5	***	1.5	***	1	ns	25.2	39.3	39	1.6	***	1.5	***	1	ns
Detailed	Lung Ca	11.2	21.2	17.9	1.9	***	1.6	***	0.8	ns	19.3	35.6	28.4	1.8	***	1.5	***	0.8	***
	Colorectal Ca	8.5	12	8.9	1.4	*	1	ns	0.7	bl	9.7	11.7	9.9	1.2	*	1	ns	0.8	*
	Lip-Or.Cav-Phar.Ca	1.3	0.7	1.5	0.6	ns	1.2	ns	2	ns	1.6	2.6	2.5	1.6	*	1.6	***	0.9	ns
	LiverCancer	1.3	2.2	1.1	1.6	ns	0.8	ns	0.5	ns	2	2.6	2.4	1.3	ns	1.2	*	0.9	ns
	Breast Ca	32.6	36.3	30.2	1.1	ns	0.9	ns	0.8	*	33.7	37.7	31.4	1.1	*	0.9	**	0.8	***
	Isc.Heart Dis.	4.4	8.4	6.9	1.9	**	1.6	***	0.8	ns	12.5	17.9	17.8	1.4	***	1.4	***	1	ns
	Cer.vasc.Dis/HTA	6.2	8.7	8.1	1.4	bl	1.3	*	0.9	ns	11.9	16.2	15	1.4	***	1.3	***	0.9	ns
	C.o.p.d.	1.6	3.4	4	2.2	*	2.6	***	1.2	ns	5.9	10.4	13	1.8	***	2.2	***	1.2	**
	Alc. Rel Dth	5.6	8.7	10.7	1.6	*	1.9	***	1.2	ns	7.4	16.5	18.1	2.2	***	2.5	***	1.1	ns
	Diabetes	0.8	3.1	2.2	3.9	*	2.9	***	0.7	ns	2.2	4.5	4.3	2.1	***	2	***	1	ns
	Mental/neurol. Dis	7.8	7.4	10.4	0.9	ns	1.3	**	1.4	*	10.7	14.5	18.1	1.4	***	1.7	***	1.2	**
	Ill Defined	3.4	5.8	5.2	1.7	*	1.5	**	0.9	ns	3.9	9	9.3	2.3	***	2.4	***	1	ns
	Suicide	12.6	20.5	16.1	1.6	***	1.3	**	0.8	*	14.2	23.6	18.3	1.7	***	1.3	***	0.8	***
	Transport Acc.	3	2.6	5.4	0.9	ns	1.8	***	2	**	4.2	2.7	7.7	0.7	*	1.8	***	2.8	***

Note: Rates are expressed per 100,000 person-years
